# Successful pregnancy in a cystic fibrosis patient with a severe impairment of lung function receiving Elexacaftor-Tezacaftor-Ivacaftor

**DOI:** 10.1016/j.rmcr.2022.101776

**Published:** 2022-11-08

**Authors:** Zisis Balmpouzis, Annabelle Faure van Rossum, David Baud, Alice Panchaud, Georgia Mitropoulou, Jesica Mazza Stalder, Angela Koutsokera

**Affiliations:** a Adult Cystic Fibrosis and CFTR-related Disorders Center, Division of Pulmonology, Dept of Medicine, Lausanne University Hospital and University of Lausanne, Switzerland; b Lung Transplant Center, Division of Pulmonology, Dept of Medicine, Lausanne University Hospital and University of Lausanne, Switzerland; c Operative Gynecology and Obstetrics, Private Practice, Switzerland; d Materno-fetal and Obstetrics Research Unit, Department Woman-Mother-Child, Lausanne University Hospital, 1011, Lausanne, Switzerland; e Institute of Primary Health Care (BIHAM), University of Bern, Bern, Switzerland; f Service of Pharmacy, Lausanne University Hospital and University of Lausanne, Lausanne, Switzerland

**Keywords:** Cystic fibrosis, Impaired lung function, Pregnancy, Elexacaftor-Tezacaftor-Ivacaftor

## Abstract

Before the arrival of Cystic Fibrosis Transmembrane Conductance Regulator (CFTR) modulators women with CF and impaired lung function were experiencing a high risk of complications and mortality during and the years after pregnancy. The arrival of the highly efficient CFTR modulator, Elexacaftor-Tezacaftor-Ivacaftor (ETI) resulted in an improvement of lung function, quality of life and fertility. Here we report a case of successful pregnancy and uncomplicated delivery for a CF patient with severely impaired lung function receiving ETI prior to conception.

## 1 Introduction

In individuals with cystic fibrosis (CF), mutations in the Cystic Fibrosis Transmembrane Conductance Regulator (CFTR) gene and subsequent dysfunction of the CFTR protein lead to defective transport of chloride, bicarbonate ions and water across epithelial membranes. Although the respiratory and the digestive systems are affected the most, CFTR is also expressed in the reproductive system where CFTR dysfunction is associated with male infertility and female subfertility [1].

In women with CF, the reproductive system anatomy is preserved, however, CFTR is expressed in the cervix, the endometrium and the fallopian tubes [1]. CFTR dysfunction results in thicker cervical mucus hampering sperm penetration and creates a pH-imbalanced environment in the uterus affecting sperm capacitation and fertilization [2]. Older maternal age and exocrine pancreatic insufficiency are also risk factors for subfertility in women with CF [3]. Women with CF having a moderate-to-severe lung function impairment, defined as a forced expiratory volume in 1 s (FEV1) lower than 50% of the predicted value (pFEV1%), carry a higher risk of delivery by cesarean section and giving birth to infants with lower birth weight [4]. Ashcroft et al. [5] also found a positive correlation between lower FEV1 and the risk for preterm delivery and giving birth to infants with lower birth weight for the gestational age. The effect of pregnancy on maternal lung function remains controversial; recent studies did not find any difference in lung function decline and maternal survival following pregnancy [4,6], on the contrary an Italian study found a lung function (FEV1 and forced vital capacity) and body mass index (BMI) decline the year following pregnancy [7]. Nevertheless the recently published expert recommendations for pregnancy in CF, suggest an optimization of lung function and ideally a pFEV1 higher than 60% before pregnancy [6].

CFTR modulators are specifically designed to address the CFTR protein defect. Elexacaftor-Tezacaftor-Ivacaftor (ETI), a highly effective CFTR modulator combination, is associated with an improvement in lung function and nutritional status, a decreased rate of pulmonary exacerbations and improved quality of life [8]. In addition, CFTR modulators seem to also improve fertility in female CF patients probably through modification of the consistency of cervical mucus and uterine fluids. Improved fertility and, consequently, unplanned pregnancies have been reported in women receiving CFTR modulator treatment [9]. Although animal data did not show any teratogenic or fetotoxic effects [10], and a series of 45 pregnancies of women receiving ETI seems reassuring [11], the currently available human safety data do not allow a reproductive risk assessment. Conversely, the little available evidence suggests effectiveness of the combined preparation for maternal health during pregnancy; 5 out of 6 mothers that stopped the CFTR modulators due to safety concerns for the fetus experienced a respiratory deterioration [11].

Here, we present a case of a successful pregnancy and vaginal delivery in a CF patient with severe lung function impairment (pFEV1 40%) treated with ETI. This is the first case of pregnancy while on ETI in Switzerland and the first detailed description of a pregnancy in a patient receiving ETI with such low FEV1 levels.

## 2 Case presentation

This is a 30-year old individual with CF having a Phe508del/Y1092X genotype, diffuse bronchiectasis colonized by *Pseudomonas aeruginosa* and exocrine pancreatic insufficiency. In 2017, following an infectious exacerbation, the patient experienced an important pulmonary function decline (nadir FEV1 at 0.63 L, 23% of the predicted value) and had severe malnutrition (BMI 16 kg/m^2^). At that time, the transthoracic echocardiogram (TTE) showed signs of pulmonary hypertension (dilated right ventricle and an estimated systolic pulmonary artery pressure of 50 mmHg) and a patent foramen ovale. These results motivated a discussion for lung transplantation, an option the patient declined. Over the following years, FEV1 remained stable at 0.8 L (30% of predicted), but the patient's condition was precarious especially during infectious exacerbations. On February 2019, she presented with her lowest recorded value of FEV1 at 0.57L (21% of predicted), before receiving antibiotic treatment for an infectious exacerbation. In August 2020, ETI was initiated through the compassionate access program before its official approval in Switzerland on February 1, 2021. Following ETI initiation, the patient experienced a marked improvement of her general status and respiratory symptoms. The FEV1 increased up to 1.09L (40% of predicted) one month after starting ETI, nutritional status improved (BMI 18.2 kg/m2) and, since ETI start, the patient did not present any exacerbations requiring systemic antibiotics.

An unplanned pregnancy occurred in December 2021, around 4 months after ETI initiation, this being the first pregnancy for this patient. After a detailed genetic workup of the couple and following an extensive discussion about the risks of maternal and fetal morbidity and mortality in this setting, the patient decided to continue with the pregnancy. Despite the current scarcity of reproductive safety data, we pursued ETI considering the risk for maternal deterioration upon treatment cessation [11].

**Fig. 1:**
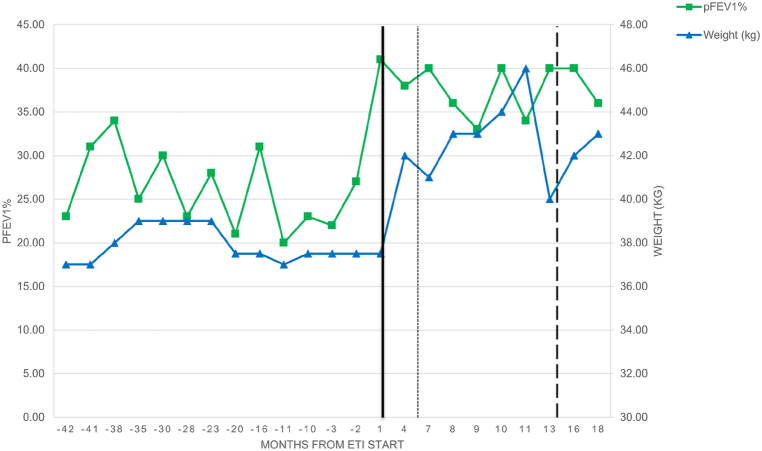
Evolution of pulmonary function tests and weight over time Abbreviations: ETI: elexacaftor, tezacaftor, ivacaftor; pFEV1%: forced expiratory volume in 1 s as percentage of the predicted value. The time-point zero refers to time of ETI initiation (black vertical line). Time-points after ETI start are noted as positive months and time-points before ETI start as negative months.  line indicates the estimated time of conception line indicates the time of delivery.

Close obstetric follow up confirmed normal fetal anatomy, growth and development over the course of pregnancy. Before the 31st week, the maternal pFEV1 remained between 35 and 40%, relatively stable compared to the values obtained immediately before the pregnancy (Fig. 1). Up to this time, the patient had not developed resting or nocturnal hypoxemia but the 6-min walking test showed exertional desaturation. In contrast to the TTE performed in summer 2017, the TTE carried out on the 13th and on the 31st week of gestation did not show a patent foramen ovale nor signs of pulmonary hypertension. There was no argument for allergic bronchopulmonary aspergillosis (ABPA) at any time-point, the patient did not develop gestational diabetes and an iron deficiency with mild anemia was substituted intravenously (IV) at the 31st and the 32nd week of pregnancy.

The course was uncomplicated until the 31st week when the patient presented symptoms of threatened preterm labour. She was hospitalized at the obstetric ward and received atosiban, followed by nifedipine for tocolysis and betamethasone for fetal lung maturation with a favorable evolution. At the same time, she contracted a rhinovirus infection causing a productive cough and bronchial congestion. A new oximetry showed nocturnal desaturation necessitating oxygen supplementation during sleep. The frequency of respiratory physiotherapy sessions was increased and inhaled formoterol/budesonide was also started. The patient refused concomitant antibiotic therapy and returned home after one week of hospital stay.

On the 33rd week of gestation, she developed symptomatic polyhydramnios treated with amniocentesis and drainage of 2700 ml of amniotic fluid. After a surveillance period of 12 hours post drainage, the patient was able to return home.

On the 36th week of pregnancy, she developed a spontaneous rupture of the membranes and gave birth to a healthy infant of 2.7 kg through vaginal delivery. To date, 12 months after delivery, the infant growth and development are uncomplicated. After childbirth, the nocturnal hypoxemia of the mother corrected rapidly and oxygen therapy was stopped. Four months after delivery, the FEV1 increased to 1.07L, 40% of the predicted value, at a similar level as before pregnancy (Fig. 1). The patient has also regained her baseline functional status.

## 3 Discussion

Our case highlights the new challenges and opportunities clinicians and individuals with CF may face in the era of highly effective CFTR modulators regarding fertility and pregnancy. Considering that the majority of CF women receiving ETI are of childbearing age, family planning and contraception should be an integral part of the CF consultation before initiation of modulators and should be discussed regularly thereafter. Clear communication in this regard is essential to decrease the risk of unplanned pregnancies and to allow informed decision making for planned pregnancies. Furthermore, the improvements in quality of life, pulmonary function and the lower risk of exacerbations during treatment with highly effective CFTR modulators may allow childbearing to be (re)considered by more CF women with decreased pulmonary function [12]. However, data about maternal mortality and morbidity as well as information about the short- and long-term safety of these treatments for the child remain scarce. Registries including fertility, fetal and maternal outcomes will prove essential to improve our understanding and to optimize patient care in the new landscape of highly effective CFTR modulators.

## 4 Conclusion

The arrival of ETI has brought unprecedented changes to the CF care. The clear improvement of respiratory and nutritional status and increased fertility have changed family planning and childbearing decisions for CF women receiving this treatment. Following pregnant CF patients receiving ETI will be an increasingly frequent challenge that CF clinicians and obstetricians will face in the following years.

## Statement of ethics

The patient provided written informed consent for publication (available upon request).

## Funding sources

No funding was received for this case report.

## Author contribution

ZB collected the data and wrote the manuscript. AK and JMS revised the manuscript. All authors contributed in patient follow-up, critically revised the manuscript and approved the final version.

## References

[1] Tizzano E., Silver M., Chitayat D., Benichou J.-C., Buchwald M. (1994). Differential cellular expression of cystic fibrosis Transmembrane regulator in human reproductive tissues, clues for the infertility in patients with cystic fibrosis. Am. J. Pathol..

[2] Ahmad A., Ahmed A., Patrizio P. (2013). Cystic fibrosis and fertility. Curr. Opin. Obstet. Gynecol..

[3] Shteinberg M., Lulu A.B., Downey D.G., Blumenfeld Z. (2019). Failure to conceive in women with CF is associated with pancreatic insufficiency and advancing age. J. Cyst. Fibros..

[4] Reynaud Q., Rousset Jablonski C., Poupon-Bourdy S. (2020). Pregnancy outcome in women with cystic fibrosis and poor pulmonary function. J. Cyst. Fibros..

[5] Ashcroft A., Chapman S.J., Mackillop L. (2020). The outcome of pregnancy in women with cystic fibrosis: a UK population-based descriptive study. BJOG.

[6] Jain R., Kazmerski T.M., Zuckerwise L.C. (2022). Pregnancy in cystic fibrosis: review of the literature and expert recommendations. J.Cyst.Fibrosis..

[7] Giordani B., Quattrucci S., Amato A., Salvatore M., Padoan R. (2018). A case-control study on pregnancy in Italian Cystic Fibrosis women. Data from the Italian Registry. Respir. Med..

[8] Middleton P.G., Mall M.A., Drevinek P., Lands L.C., McKone E.F., Polineni D. (2019). Elexacaftor-Tezacaftor-Ivacaftor for cystic fibrosis with a single Phe508del Allele. NEJM.

[9] Taylor-Cousar J.L. (2020). CFTR modulators: impact on fertility, pregnancy, and lactation in women with cystic fibrosis. J. Clin. Med..

[10] FDA. Highlights of Prescribing Information for TRIKAFTA™ (Elexacaftor, Tezacaftor and Ivacaftor Tablets; Ivacaftor Tablets), Co-packaged for Oral Use.

[11] Taylor-Cousar J.L., Jain R. (2021). Maternal and fetal outcomes following elexacaftor-tezacaftor-ivacaftor use during pregnancy and lactation. J.Cyst.Fibrosis..

[12] Kazmerski T.M., Gmelin T., Slocum B., Borrero S., Miller E. (2017). Attitudes and decision making related to pregnancy among young women with cystic fibrosis. Matern. Child Health J..

